# The conundrum of neuropsychiatric systemic lupus erythematosus: Current and novel approaches to diagnosis

**DOI:** 10.3389/fneur.2023.1111769

**Published:** 2023-03-21

**Authors:** Jonathan S. Emerson, Simon M. Gruenewald, Lavier Gomes, Ming-Wei Lin, Sanjay Swaminathan

**Affiliations:** ^1^Department of Clinical Immunology and Immunopathology, Westmead Hospital, Sydney, NSW, Australia; ^2^Sydney Medical School, The University of Sydney, Sydney, NSW, Australia; ^3^Centre for Immunology and Allergy Research, Westmead Institute for Medical Research, Sydney, NSW, Australia; ^4^Department of Nuclear Medicine, PET and Ultrasound, Westmead Hospital, Sydney, NSW, Australia; ^5^Department of Radiology, Westmead Hospital, Sydney, NSW, Australia; ^6^Department of Clinical Immunology, Blacktown Hospital, Sydney, NSW, Australia; ^7^School of Medicine, Western Sydney University, Sydney, NSW, Australia

**Keywords:** systemic lupus erythematosus, central nervous system vasculitis, brain diseases, biomarkers, autoantibodies, neuroimaging (anatomic and functional)

## Abstract

Recognising neuropsychiatric involvement by systemic lupus erythematosus (SLE) is of growing importance, however many barriers to this exist at multiple levels of our currently available diagnostic algorithms that may ultimately delay its diagnosis and subsequent treatment. The heterogeneous and non-specific clinical syndromes, serological and cerebrospinal fluid (CSF) markers and neuroimaging findings that often do not mirror disease activity, highlight important research gaps in the diagnosis of neuropsychiatric SLE (NPSLE). Formal neuropsychological assessments or the more accessible screening metrics may also help improve objective recognition of cognitive or mood disorders. Novel serum and CSF markers, including autoantibodies, cytokines and chemokines have also shown increasing utility as part of diagnosis and monitoring, as well as in distinguishing NPSLE from SLE patients without SLE-related neuropsychiatric manifestations. Novel neuroimaging studies also expand upon our existing strategy by quantifying parameters that indicate microarchitectural integrity or provide an assessment of neuronal function. Some of these novel markers have shown associations with specific neuropsychiatric syndromes, suggesting that future research move away from considering NPSLE as a single entity but rather into its individually recognized neuropsychiatric manifestations. Nevertheless, it is likely that a composite panel of these investigations will be needed to better address the gaps impeding recognition of neuropsychiatric involvement by SLE.

## 1. Introduction

Systemic lupus erythematosus (SLE) is an autoimmune disease that may affect multiple organs and often presents heterogeneously between individuals. While it more commonly involves the cutaneous, musculoskeletal, serosal and renal systems, despite the initial descriptions of neurological symptoms in SLE patients during the late 19^th^ century, there is still potential to address the many gaps in our understanding of neuropsychiatric involvement of SLE (NPSLE). The prevalence rates of NPSLE vary widely in the published literature, estimated to be between 12 and 95%, which may partly be owing to the variability of NPSLE definitions used, study designs, study populations and ethnicities included, amongst other factors (1). Furthermore, numerous factors hinder recognition and diagnosis of NPSLE, including the heterogeneity of neurological symptoms, the absence of standardized assessment, the unreliability of conventional markers for diagnosis and monitoring of disease, as well as a paucity of good quality evidence for its effective treatment. Although the true impact of NPSLE would be difficult to estimate due to these factors, studies have demonstrated higher rates of morbidity and mortality in those with NPSLE amongst SLE cohorts (2, 3).

This review opens with a case that highlights the complexities involved in the current diagnosis and monitoring of NPSLE, and proposes potential novel additions that may help overcome these challenges.

## 2. Case

A female in her late teens presented to hospital with polyarthralgias, myalgias, and nephrotic syndrome. Bloods revealed an elevated CRP of 20 mg/L, ESR of 120 mm/h, hypocomplementemia, speckled anti-nuclear antibody (ANA) titer of 1:2560, normal anti-dsDNA antibody levels and negative extractable nuclear antigen (ENA) and antiphospholipid antibody profile. Renal biopsy demonstrated class V lupus nephritis. She was commenced on oral corticosteroids, hydroxychloroquine, mycophenolate and tacrolimus, with symptomatic improvement and normalization of her proteinuria.

Over the next 12–16 months, she experienced several episodes of depression and mania, and was commenced on sertraline and quetiapine. One year later she presented with psychosis. Bloods revealed normal inflammatory markers, complement levels, anti-dsDNA antibody levels and antiphospholipid antibody levels. CSF was acellular, had normal protein levels, and no oligoclonal bands. A magnetic resonance imaging (MRI) brain was normal, whilst the cerebral nuclear medicine single photon emission computed tomography (NM-SPECT) revealed asymmetrical areas of reduced perfusion in the frontal, parietal, and occipital lobes, and the basal ganglia, all of which were thought to favor NPSLE. She did not improve despite pulsed intravenous methylprednisolone, rituximab and co-treatment with aripiprazole, and was therefore treated with 6 once-monthly cycles of cyclophosphamide. Her psychosis improved and she was continued on tapering oral steroids, hydroxychloroquine, mycophenolate and sirolimus. Her aripiprazole was tapered and ceased over a 6-month period.

Twelve months later, she represented with mania. Investigations were again unremarkable, except for a cerebral NM-SPECT which revealed symmetrical areas of hypoperfusion, after which she was treated with rituximab, aripiprazole and olanzapine. Upon improvement, her oral corticosteroids, olanzapine and aripiprazole were tapered and ceased over a 3-6-month period. A serial cerebral NM-SPECT scan showed improvement in the perfusion deficits. She remains well and has since been able to recommence her University studies, with the plan to routinely administer rituximab every 6 months.

This case highlights a major conundrum which plagues this illness – what tools, if any, can help diagnose and monitor the activity of NPSLE?

## 3. Shortcomings of the current diagnostic algorithm

One of the main shortcomings pertaining to diagnosing SLE is the lack of objective consensus of what constitutes its diagnosis, particularly given the controversy as to whether SLE truly is a single disease or a constellation of different syndromes with differing underlying disease processes. As such, studies have investigated the value of different classification criteria, serum and CSF markers and neuroimaging studies in classifying SLE and its involvement of the central nervous system (CNS).

### 3.1. Clinical features

#### 3.1.1. The classification criteria for SLE

Various iterations of classification criteria have been developed and have aimed to classify what are essentially different clusters of clinical phenotypes within the umbrella of ‘SLE’ for investigative purposes. However, due to the absence of much needed diagnostic criteria, such classification criteria have been improperly adopted as surrogate diagnostic criteria. With this in mind, utilization of the different classification criteria to-date poses a challenge in their differing performances for the classification of SLE as well as neuropsychiatric involvement.

The first of these was the 1971 Preliminary Criteria for the Classification of SLE, which required at least 4 of 14 laboratory or clinical criteria for the classification of SLE (4), however was limited by the exclusion of immunological laboratory criteria. The subsequent 1982 Revised Criteria for the Classification of SLE, and its update, the 1997 American College of Rheumatology (ACR) Criteria, incorporated non-obligatory immunological criteria, such as the presence of an ANA, anti-DNA, anti-Smith or antiphospholipid antibodies, which may have contributed to its higher sensitivity and specificity than the 1971 classification criteria (5).

The 2012 Systemic Lupus International Collaborating Clinics (SLICC) SLE Classification Criteria differed from its predecessors by the obligatory presence of at least one laboratory and one clinical criterion, or alternatively allowed for the classification of SLE in the presence of a positive ANA or anti-DNA if there was a biopsy-proven nephritis (6). This classification criteria demonstrated better sensitivity, although lower specificity, than the 1997 ACR Criteria.

Finally, the most recent European League Against Rheumatism (EULAR) / ACR 2019 Classification Criteria differs by the obligatory presence of an ANA at titer of at least 1:80, as well as the presence of a minimum number of weighted laboratory and clinical criteria for the classification of SLE (7). This criteria has demonstrated a higher and similar sensitivity than the 1997 ACR Criteria and 2012 SLICC Criteria, respectively, and comparable specificity (8).

Whilst each of these classification criteria included either psychosis, seizures and/or delirium as part of the neuropsychiatric criterion, the 2012 SLICC Classification Criteria expanded upon these features with the addition of mononeuritis multiplex, myelitis, peripheral or cranial neuropathies and acute confusional states. As such, while this classification criteria appears to have a comparable classification performance for SLE to the EULAR/ACR 2019 Classification Criteria, it’s broader definitions of neuropsychiatric phenomena may also improve its sensitivity for NPSLE. Nevertheless, neither of these classification criteria encompass the breadth of syndromes that may characterize CNS involvement by SLE.

#### 3.1.2. ACR case definitions of neuropsychiatric syndromes

Neuropsychiatric manifestations are heterogeneous, some of which may be subtle or indistinguishable from non-SLE-related presentations, which may therefore delay diagnosis. In 1999 the ACR developed a classification with case definitions of neuropsychiatric syndromes – including 12 CNS and 7 peripheral nervous system (PNS) syndromes, possible non-SLE-related clinical associations and exclusions – which have better facilitated clinical identification of such syndromes amongst SLE cohorts (Table 1) (9). Studies have further subcategorized these syndromes as ‘diffuse’ or ‘focal’ manifestations (10), which may facilitate disease prognostication, as demonstrated by a study of 68 SLE patients in which those with diffuse, but not focal, NPSLE manifestations showed higher disease activity than those with non-SLE-related neuropsychiatric manifestations (11). Incorporation of these case definitions of neuropsychiatric syndromes with the above classification criteria for SLE may, therefore, prove a better way for classification of neuropsychiatric involvement by SLE.

**Table 1:** ACR (1999) case definitions for neuropsychiatric syndromes, further separated into diffuse and focal syndromes (9, 10).

Diffuse Syndromes	
Syndromes associated with the CNS	Aseptic meningitis
	Acute confusional state
	Cognitive dysfunction
	Demyelinating syndrome
	Headache
	Psychosis
	Mood disorder
	Anxiety disorder
Focal Syndromes	
Syndromes associated with the CNS	Cerebrovascular disease
	Seizures
	Myelopathy
	Movement disorder
Syndromes associated with the PNS	Acute inflammatory demyelinating polyradiculoneuropathy (Gullain-Barre syndrome)
	Cranial neuropathy
	Mononeuropathy
	Myasthenia gravis
	Plexopathy
	Autonomic neuropathy
	Polyneuropathy

Despite these definitions, the prevalence of NPSLE varies widely between studies, and arguably lacks specificity, given the inclusion of syndromes that are common in the general population. Therefore, attributing such identified syndromes to SLE or an alternative cause has also become a major challenge, for which these development of different algorithms have attempted to mitigate.

#### 3.1.3. Attribution models for the diagnosis of NPSLE

Monov and Monova attempted to define an algorithm for diagnosing NPSLE, part of which was based on the presence of a minimum set of criterion including specific neuropsychiatric manifestations and/or additional investigation findings, with high sensitivity (90.3%) and moderate specificity (67.7%) (12). This, however, did not encompass all of the CNS or PNS syndromes outlined by the 1999 ACR case definitions for neuropsychiatric syndromes, nor did consider the influence of confounding factors to SLE attribution.

In 2007, two models using the SLICC inception cohort of newly diagnosed SLE individuals were proposed for attributing the presence of neuropsychiatric manifestations to their underlying SLE, and were based on three factors – the temporal association of the neuropsychiatric syndrome with SLE onset, whether or not the neuropsychiatric syndrome was considered as a minor or non-specific event (13), and the presence of either non-SLE-related clinical associations or exclusions that could have contributed to the event (14). Both models, designated as ‘A’ and ‘B’, differed in terms of the stringency of the temporal association between the neuropsychiatric syndrome and SLE diagnosis and the presence or absence of any non-SLE-related clinical associations. The more stringent model (‘A’) included those with onset of neuropsychiatric manifestations within 6 months prior to the SLE diagnosis and the absence of any non-SLE-related associations or exclusions, and displayed a sensitivity and specificity of 23 and 96%, respectively; whereas the less stringent model (‘B’) included those with onset within 10 years and the absence of any non-SLE-related exclusions but not associations, and displayed a sensitivity and specificity of 35 and 79%, respectively (15).

In 2015, the Italian Society of Rheumatology expanded upon the SLICC attribution models and developed a point-based algorithm for attributing a neuropsychiatric syndrome to SLE based on four weighted factors – all of the same factors from the SLICC model, with the addition of the presence of factors that favored SLE – which demonstrated the best combination of sensitivity and specificity of 87.9 and 82.6%, respectively (16, 17).

Therefore, incorporation of such attribution models in conjunction with the 1999 ACR case definitions for neuropsychiatric syndromes may better facilitate recognition of NPSLE cohorts and distinguish these from those without neuropsychiatric involvement.

### 3.2. Serum

The 2019 EULAR/ACR classification criteria for SLE and the SLE Disease Activity Index (SLEDAI) incorporate a number of serological criteria for the classification of SLE and stratification of its activity, respectively (7, 18). These, however, often cannot be used to predict neuropsychiatric disease activity in the absence of concurrent systemic inflammation (19).

#### 3.2.1. Anti-dsDNA antibodies

While anti-dsDNA antibodies are highly specific for SLE and tend to correlate with disease activity (20), they have limited utility in isolated neuropsychiatric involvement. They may be found in only 70% of NPSLE patients, and levels do not appear to correlate with neuropsychiatric disease activity (19, 21). Further complicating this are the diverse methods used to measure anti-dsDNA antibody levels, each of which differ in their diagnostic performance and produce results that do not necessarily correlate between methods (22). Therefore, whether its unreliability in NPSLE is truly due to pathophysiological differences from non-neuropsychiatric SLE, or whether due to differences in laboratory method performance is uncertain, and further studies using more homogeneous methods of laboratory assessment are required.

#### 3.2.2. The extractable nuclear antigens

Anti-ribosomal P antibodies have a prevalence of 10–47% in SLE, and tend to occur more commonly in paediatric- than adult-onset SLE (23). A meta-analysis demonstrated its association with NPSLE, particularly for psychosis and depression (pooled odds ratios [OR] of 3.08 and 3.03, respectively) (23). Similar to anti-dsDNA antibodies, the different diagnostic assays utilized may influence diagnostic performance, which was highlighted in a recent meta-analysis and may explain large variations in reported prevalence rates as well as inconsistently reported associations with NPSLE (23). Specifically, utilization of indirect immunofluorescence (IIF)-based assays, as used for ANAs, may not uncommonly be falsely reported as ‘negative’ due to inexperience in identifying the characteristic cytoplasmic fluorescence, laboratory policies avoiding reporting cytoplasmic patterns, and sensitivity of substrates used for IIF-based detection. Additionally, despite better sensitivities, modern solid phase assays may not always routinely test for anti-ribosomal P antibodies, and have also shown poor inter-method correlations (24). It is, therefore, important to clarify ENA testing algorithms when assessing for anti-ribosomal P antibodies.

Anti-Smith antibodies are specific for SLE and tend to associate with more severe manifestations such as renal disease, vasculitis, and haemolytic anaemia, as well as disease activity (25, 26). Further, a large cohort study demonstrated associations with neurologic disorders, seizures and psychosis (adjusted ORs of 1.66, 1.44 and 1.82, respectively) (25), all of which may also be supported by the observation of serum titer correlation with markers of blood–brain barrier permeability (27) and association of seropositivity with reduced grey matter density on MRI (28).

While no definite associations have been established with anti-SSA/Ro or anti-SSB/La antibodies, a few studies have suggested possible associations with NPSLE, including an association between seropositivity for anti-SSA antibodies and severe neuropsychiatric damage (26), and of reduced white matter density on MRI (28). Interestingly, a study of patients with neuromyelitis optica spectrum disorder, including those with and without SLE, also demonstrated an association of anti-SSB antibodies with disease activity and disability, which may suggest independent mechanisms that influence neuropsychiatric involvement and may warrant further investigation (29).

#### 3.2.3. Antiphospholipid antibodies

aPLAs consist of the anti-cardiolipin (aCL) and anti-beta-2-glycoprotein I antibodies (aβ2GPI), and the lupus anticoagulant (LAC), which may cause disease by processes that culminate in thromboembolic phenomena, which may underpin their role in NPSLE. They may be found in 30–50% of SLE patients, up to half of whom may go on to develop features of the antiphospholipid antibody syndrome, and may also present with both focal or diffuse NPSLE manifestations (30, 31). Cognitive disorders have been reported in 54% of aPL-positive SLE patients, compared to 7% of those seronegative (32), and has shown associations with aCL or LAC (33). Mood disorders in SLE have shown associations with aβ2GPI, and seizure disorders and acute confusional states with aCL (33).

#### 3.2.4. Conventional markers of disease activity

Inflammatory markers are typically elevated in patients with systemic rheumatic diseases. CRP, however, is only elevated in around 30% of patients with treatment-naïve SLE (34). Although CRP and ESR levels may increase with active musculoskeletal disease (35), these may be normal in NPSLE patients. Similarly, while reduced serum complement levels may typically accompany active SLE, it may only be associated with certain neuropsychiatric manifestations and was even demonstrated to be normal in 66% of patients with active NPSLE (19, 21).

Therefore, although certain serum markers have demonstrated associations with SLE, they are largely non-specific, are measured using different assays with variable diagnostic performances, do not portend neuropsychiatric involvement, and not uncommonly remain quiet during active disease. They are thus of minimal utility in NPSLE, and better surrogates reflecting CNS pathology have been pursued.

### 3.3. Cerebrospinal fluid

Although necessary to exclude other aetiologies, CSF findings may also be non-specific. A pleocytosis has been reported in around 20% of NPSLE cases and is typically of low-level although has been reported with white cell counts greater than 100 cells/μl (21, 36). Protein elevation may be seen in 20–30% of cases, with levels around 1 g/L, although may increase to greater than 2 g/L (37, 38). The presence of oligoclonal bands and an elevated IgG/albumin index, which are suggestive of intrathecal IgG synthesis, have been reported in up to 42% of NPSLE cases (39), particularly amongst those with diffuse or complex, in contrast to focal, neuropsychiatric presentations (40). While associated with a worse prognosis in NPSLE, CSF abnormalities have been reported in only around 40% of cases, and therefore do not provide a reliable discrimination of NPSLE from non-neuropsychiatric SLE patients (37, 38).

### 3.4. Neuroimaging

**Figure 1:**
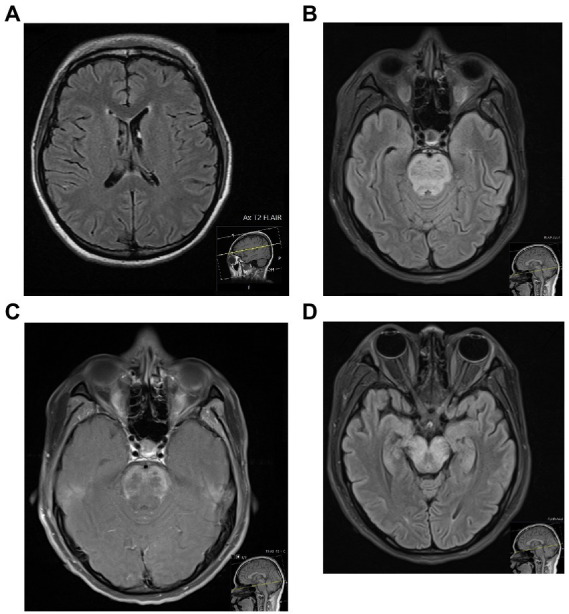
Two patients with NPSLE, who both presented with seizures in the context of a systemic hyperinflammatory syndrome. The transaxial MRI FLAIR of the first patient **(A)**, a 27-year old female, was unremarkable. The MRI of the second patient **(B–D)**, a 20-year old female, showed diffuse swelling of the pons with increased T2/FLAIR signal **(B)** and diffuse peripheral abnormal contrast enhancement **(C)**, with extension of the T2/FLAIR signal inferiorly to the lower medulla and superiorly across white matter tracts of the midbrain **(D)** – all of which were in keeping with a severe CNS vasculitis.

Neuroimaging by conventional MRI (cMRI) plays an important role in the workup of NPSLE. These have demonstrated various pathologies, including atrophy, demyelination, and ischaemic, haemorrhagic or inflammatory lesions (41). Findings consistent with small vessel disease have been frequently reported in newly diagnosed NPSLE patients – including white and grey matter lesions, atrophy, microbleeds, and lacunes – followed by large vessel disease, and least commonly, inflammatory lesions (42). Findings do not appear to correlate with SLEDAI (43), anti-dsDNA antibody or complement levels, nor CSF parameters (44). It is possible that these changes reflect chronic, incompletely controlled disease, by which time such findings may be partially irreversible independent of treatment, therefore further complicating recognition of active NPSLE. Abnormalities, however, have been reported in only around 20 to 70% of NPSLE patients (Figure 1) (41, 44), and therefore again are unable to provide a highly sensitive way to exclude CNS involvement of SLE.

## 4. Potential additions to current diagnostic algorithms

Therefore, the limitations posed by relying on conventional methods of diagnosing NPSLE has prompted the need to expand our diagnostic tools, including neuropsychological assessment and novel serum, CSF and neuroimaging investigations.

### 4.1. Neuropsychological testing

Neuropsychiatric syndromes such as cognitive or mood disorders may be difficult to recognize without formal, structured assessment. Studies that have incorporated these assessments have actually reported higher prevalence rates of NPSLE than those that made unstructured assessments on symptomatic patients (13, 14, 45), thus highlighting a role in identifying subclinical manifestations, and potential to monitor therapeutic efficacy in those with reversible disease processes.

#### 4.1.1. Cognitive impairment

Comprehensive neuropsychological testing batteries (CBs), considered as gold standard, reported prevalences of cognitive impairment in around 40% of all SLE and 80% of specifically NPSLE patients – although such estimates may be inaccurate due to variable definitions of cognitive impairment and NPSLE used across studies (46). Nevertheless, the significant time, cost and training requirements imposed by CBs, as well as the subsequently derived Automated Neuropsychologic Assessment Metrics (ANAM) and the SLE-validated ACR-SLE batteries, may hinder routine administration of these tools by healthcare professionals (47, 48). Hence, screening metrics for cognitive impairment may be considered as acceptable surrogates.

Many have been validated for use in SLE populations, although may either test specific cognitive domains or are susceptible to biases. For example, the Hopkins Verbal Learning Test-Revised (HVLT-R) and the Controlled Oral Word Association Test (COWAT) measure verbal learning and recall, and verbal fluency, respectively, and show only moderate sensitivities and specificities compared to the ACR-SLE battery (49). Self-report screening questionnaires, such as the Cognitive Symptom Inventory (CSI), have shown lower sensitivities for cognitive impairment and may be influenced by patients’ self-awareness of cognitive deficits and underlying mood disorders – which also compromise their reliability (50, 51).

In contrast, the Mini Mental State Examination (MMSE) and Montreal Cognitive Assessment (MoCA) are clinician-administered metrics that assess multiple cognitive domains and only take 5–10 min to administer. While both show moderate-to-high specificities for cognitive impairment in SLE patients, the MoCA has demonstrated a higher sensitivity (52). Either may therefore prove useful given their brevity, simple administration, and the lack of cost nor need for special training to administer.

#### 4.1.2. Mood disorders

Gold standard testing for mood disorders involves clinical interviews using the Diagnostic and Statistical Manual of Mental Disorders (DSM) or International Classification of Diseases (ICD), which has shown prevalences of major depression and anxiety among SLE patients of 24 and 37%, respectively (53). This assessment, however, is also complicated by the need for specialized training, cost and time to administer. Screening metrics for depression and anxiety in SLE patients include the Centre for Epidemiological Studies Depression Scale (CES-D), the Back Depression Inventory, Beck Anxiety Inventory, and the Hospital Anxiety and Depression Scale (HADS), amongst others. The HADS has shown a high sensitivity and specificity for anxiety, and the CES-D has shown a high sensitivity for depression (54). Although there are no head-to-head studies comparing their diagnostic performance in SLE patients, the preference for either tool will likely depend on convenience and ease of administration.

While these tools may improve detection of mood or cognitive phenomena, the influence of corticosteroid treatment should be noted, given the disparity between its therapeutic benefits and known adverse effects on neurocognitive symptoms. Additionally, cultural and educational backgrounds of the patients are also uncontrolled variables in any subjective questionnaires. More longitudinal studies will be needed to determine the impact of these factors on the outcomes of such testing.

### 4.2. Novel serum and CSF markers

The lack of laboratory markers for NPSLE has led to studies of more novel molecules and antibodies, which have produced some promising findings (Table 2).

**Table 2:** Novel biomarkers for NPSLE.

Biomarkers			NPSLE vs. non-NP SLE		All SLE vs. HC		Post-treatment
			Serum/Plasma	CSF	Serum/Plasma	CSF	
Auto-antibodies	Anti-NR2 (55–57)	Levels	ND	↑(CNS-NPSLE)			↓(Serum, CSF)
		Associations	Prevalence 25–35% in all SLE				
			Seropositivity associated with 1.6-fold higher odds of NPSLE				
	^A^Anti-UCH-L1 (58, 59)	Levels	^B^↑	↑			^B^↓ (serum)
		Associations	^B^Serum levels directly correlate with ESR, anti-dsDNA, and negatively correlate with C3				
			^B^Serum levels correlate with SLE disease activity				
			^B^Sensitivity 37.5%, specificity 92.3% for NPSLE versus non-NP SLE (serum)				
	AECA (60, 61)	Levels			↑		
		Associations	Prevalence in serum >60% in NPSLE vs. ~30% in non-NP SLE				
			Vasculitis				
			Serum levels correlate with SLE disease activity				
	^A^Anti-MAP2 (62, 63)	Levels		↑			
		Associations	Prevalence in serum ~40% in NPSLE vs. ~6% in non-NP SLE				
			Prevalence in CSF ~30% in NPSLE				
			>70% of seropositive SLE have NPSLE				
			CSF positivity has high specificity for NPSLE				
			CSF positivity associated with higher levels of CSF anti-ribosomal P levels and IL-6 levels				
			↑CSF levels in NPSLE than non-SLE CTD				
	^A^Anti-SBSN (64, 65)	Levels	ND	↑			
		Associations	Prevalence in CSF ~40% in NPSLE				
			Sensitivity 41.9%, specificity 91.8% for NPSLE (CSF)				
			↑serum levels in NPSLE than MS & VM, ↑CSF levels in NPSLE than MS & NPH				
	^A^Anti-TPI (66)	Levels	↑				
		Associations	Possible association with aseptic meningitis				
			Sensitivity 32.3%, specificity 95% for NPSLE (serum); however, single study of 31 NPSLE patients				
Markers of BBB disruption or neural damage	^A^S100A8/A9 (67)	Levels	^C^↑/ND	ND	↑		
		Associations					
	^A^S100B (68–71)	Levels	↑	↑	↑		↓(CSF, not serum)
		Associations	Higher serum levels associated with cognitive impairment and peripheral neuropathy				
			Sensitivity 20%, specificity 65% for CNS-NPSLE (serum); however did not include minor or non-specific neuropsychiatric syndromes in this cohort				
			Elevated serum levels associated with 2.3-fold higher odds of neuropsychiatric manifestations in children with SLE				
	^A^GFAP (72)	Levels		↑(3-fold)			↓(CSF)
		Associations	Sensitivity 48%, specificity 87% for NPSLE (CSF)				
	^A^NfL (72–75)	Levels	^C^↑(association with focal CNS involvement) /ND	^C^↑(7-fold)/ND	↑		↓(CSF)
		Associations	CSF levels associated with impaired psychomotor speed and motor function				
			CSF levels correlate with CSF IL-6, IL-8, anti-NR2 levels				
			Sensitivity 74%, specificity 65% for NPSLE (CSF)				
			Higher plasma NfL levels correlate with larger total CSF volumes by MRI				
	^A^MMP-9 (76, 77)	Levels	↑	↑	ND	↑	
		Associations	CSF levels correlate with CSF IL-6, IL-8, GFAP levels				
			Associations of serum levels with cognitive impairment and T1- and T2-weighted lesions on cerebral MRI				
Cytokines/Chemokines	BAFF/APRIL (78–80)	Levels		BAFF: ND		BAFF: ↑	
				APRIL: ↑		APRIL: ↑	
		Associations	Murine/*in vitro* studies: BAFF associated with microglial activation and surface Fc receptor expression				
			CSF APRIL levels correlate with fatigue				
	^A^IL-6 (81–86)	Levels	ND	↑	↑		↓(CSF)
		Associations	Serum levels associated with SLE disease activity, anti-dsDNA seropositivity				
			↑CSF levels in NPSLE than non-SLE with CNS infection				
			↓CSF levels in NPSLE than SLE with CNS infection				
	^A^IL-8 (81–83, 87)	Levels		↑			↓(CSF)
		Associations	↓CSF levels in NPSLE than SLE with CNS infection				
	^A^OPN (88)	Levels		↑			↓
		Associations	Correlates with markers of BBB permeability (IgG index, albumin quotient)				
			Sensitivity 70%, specificity 100% for NPSLE (CSF); however, single study of 11 NPSLE and 7 non-NP SLE patients, with the latter also including 2 patients with depression				
	IFN-α (89–91)	Levels	ND				ND (CSF, serum)
		Associations	Serum association with SLE disease activity				
			CSF level associations with acute confusional state and SLE-induced psychosis				
	IFN-γ (81, 82, 92, 93)	Levels	ND	ND			
		Associations	Seropositivity associated with cerebral ischemia on MRI				
			CSF positivity associated with multiple ischemic foci				
			CSF levels associated with cerebral volume reduction				
	^A^IP-10, MIG (83, 94, 95)	Levels		↑			↓(CSF)
		Associations	Serum associations with SLE disease activity				
			CSF levels associated with lupus-related headaches				
			↑CSF MIG levels in NPSLE than non-headache NPLSE				
	Neopterin (96–98)	Levels	↑	Unknown	↑		
		Associations	Serum levels correlate with CRP, anti-dsDNA, SLE disease activity				
			↑serum levels in all SLE, even with mild disease – good sensitivity				
			Possible utility for distinguishing neuroinflammatory cause from primary psychiatric manifestations				

AECA, anti-endothelial cell antibodies; Anti-SBSN, anti-suprabasin antibodies; Anti-TPI, anti-triosephosphate isomerase antibodies; Anti-UCH-L1, anti-ubiquitin carboxyl hydrolase L1 antibodies; APRIL, a proliferation-inducing ligand; BAFF, B-cell activating factor; BBB, blood–brain barrier; cNPSLE, central NPSLE; CNS, central nervous system; CTD, connective tissue disease; GFAP, glial fibrillary acidic protein; HC, healthy control; IP-10, IFN-γ-inducible 10-kD protein; MIG, monokine induced by IFN-γ; MMP-9, matrix metalloproteinase-9; MS, multiple sclerosis; NfL, neurofilament light; NPH, normal pressure hydrocephalus; NPSLE, neuropsychiatric systemic lupus erythematosus; Non-NP SLE, non-neuropsychiatric systemic lupus erythematosus; OPN, osteopontin; VM, viral meningitis.

A Possible utility in distinguishing NPSLE from non-NP SLE.

B Association only found with autoantibody against a specific epitope of the peptide, however not demonstrated with other tested epitopes (59).

C Conflicting results in the context of differing definitions of NPSLE or study populations used within or between studies.

#### 4.2.1. Neopterin

Neopterin is a product derived from IFN-γ-activated macrophages during the cellular immune response. Higher serum levels may be found in SLE than healthy individuals and also correlate with clinical disease activity indices (96, 97). A study of 40 SLE patients demonstrated higher serum levels in those with NPSLE than non-neuropsychiatric SLE, raising the possibility of its utility in distinguishing these two groups (97). While CSF levels have not been defined in NPSLE patients specifically, it is elevated in inflammatory neurological conditions, for example the autoimmune encephalitides, and has been shown to correlate with inflammatory activity (99, 100). Studies of non-SLE inflammatory disorders, such as multiple sclerosis and HTLV-1 infection, have even demonstrated the utility of high CSF/serum ratios in distinguishing active inflammatory or infectious CNS involvement from those without CNS involvement (101, 102). Additionally, levels are unchanged during acute psychotic episodes in patients with schizophrenia, suggesting utility in distinguishing neuroinflammatory from primary psychiatric phenomena (98). Further studies in SLE populations may help determine its value in NPSLE in the future.

#### 4.2.2. Anti-NR2A/2B subunit antibodies

Anti-NR2A/2B subunit antibodies are a subset of anti-dsDNA antibodies that cross-react with epitopes on the NR2A and NR2B subunits of NMDA receptors, however not with the NR1 subunit that is targeted in anti-NMDA receptor encephalitis (103). Murine studies have demonstrated their pathogenic potential and ability to induce neuropsychiatric symptoms, however only in the presence of blood–brain barrier disruption – which may explain why around 35% of SLE patients may be seropositive independent of neuropsychiatric phenomena (55, 104, 105). Therefore, CSF, but not serum, levels correlate with CNS disease activity (106). Nevertheless, a meta-analysis demonstrated that the proportion of seropositive individuals was higher in those with NPSLE than non-neuropsychiatric SLE, thus suggesting a potential association of seropositivity with NPSLE (56).

#### 4.2.3. Anti-UCH-L1 antibodies

Ubiquitin carboxyl-terminal hydrolase L1 (UCH-L1) is a de-ubiquitination enzyme that is present in neurons, neuroendocrine cells and gonadal tissue (107), and is involved in the inhibition of proteasomal activity and homeostasis of ubiquitin monomers. It has been associated with various pathologies, including in neurodegenerative diseases, such as Alzheimer’s or Parkinson’s diseases (108). Of specific interest is the demonstration of utility of anti-UCH-L1 antibodies, which are thought to develop in response autoimmune injury to neural tissue. A study including 36 NPSLE patients demonstrated the utility of CSF levels of these antibodies to distinguish NPSLE from non-neuropsychiatric SLE (58). Interestingly, while serum levels were unable to demonstrate an association with NPSLE in this study, a subsequent study found an association with anti-UCH-L1 antibodies that were directed against specific epitopes on the UCH-L1 peptide. This study included 32 NPSLE and 40 non-neuropsychiatric SLE patients, and demonstrated elevated serum levels of these epitope-specific anti-UCH-L1 antibodies in NPSLE, particularly in those with more severe neuropsychiatric manifestations or higher SLE disease activity, as well as reduction of levels following treatment (59). While this has shown some promise, further studies with larger populations will be needed to corroborate these findings and will also be needed to determine whether such associations exist with specific neuropsychiatric manifestations.

#### 4.2.4. IL-6

Interleukin-6 (IL-6) is a cytokine that induces hepatocyte production of acute phase proteins during an inflammatory response. Serum levels have shown associations with SLE which correspond to disease activity, although do not distinguish NPSLE, non-neuropsychiatric SLE, intracranial infections, nor non-inflammatory neurological disease (81, 84). CSF levels have shown better utility, although are also known to be elevated in other neuroinflammatory diseases. They are higher in NPSLE than non-neuropsychiatric SLE cohorts, and also fall following successful treatment, thus highlighting its potential utility for monitoring CNS activity (83). Correspondingly, a higher CSF/serum IL-6 ratio in NPSLE was demonstrated in a study of 13 CNS lupus and 17 SLE without CNS lupus patients, which may reflect greater CNS rather than systemic IL-6 production (87). This study, however, grouped patients according to the presence or absence of CNS neuropsychiatric manifestations, and therefore may not be applicable in cohorts of undifferentiated (CNS and PNS) NPSLE. Although CSF levels are unable to distinguish NPSLE from other CNS inflammatory processes, it may have a role in distinguishing NPSLE from presentations such as corticosteroid-induced psychosis or other non-inflammatory psychiatric disorders (85). Additionally, multiple reports and a phase I study have demonstrated the efficacy of IL-6 blockade in refractory arthritis or serositis, emphasizing its role in the pathophysiology of SLE (109–113). Further investigation will be needed to determine whether elevated CSF levels portend a place for IL-6 blockade in the management of NPSLE.

#### 4.2.5. IFN-α, IFN-γ, IP-10, and MIG

SLE is associated with a type I interferon (IFN) signature (89). A study of 34 NPSLE patients demonstrated elevated serum and CSF IFN-α levels, however did not show any differences compared to non-neuropsychiatric SLE nor demonstrate an association with disease activity (90). The heterogeneous study population, however, consisting of both diffuse and focal manifestations of CNS NPSLE, could have limited the ability to draw any associations, particularly given that specific manifestations have shown associations with IFN-α – such as CSF levels with acute confusional state and SLE-induced psychosis, including reductions that mirror clinical improvement in the latter (90, 91). These reinforce the possibility of a role in specific neuropsychiatric manifestations, although larger studies will be needed to verify this. Additionally, the positive outcomes of the MUSE phase II trial and the TULIP-1 and TULIP-2 phase III trials of the efficacy of anifrolumab in moderate-to-severe non-neuropsychiatric SLE portend the role of type I IFN in SLE, and may strengthen the case for further exploring this pathway in NPSLE (114).

IFN-γ, a type II IFN, is also associated with SLE (115). Studies have demonstrated elevated serum and CSF levels in NPSLE, although no differences to those with non-neuropsychiatric SLE (82). Interestingly, an association between IFN-γ levels and MRI findings have also been observed, including serum and CSF levels with cerebral ischemic changes, and CSF levels with cerebral volume reduction – however larger studies will also be needed to better determine its utility in NPSLE (81, 92, 93).

IFN-γ-inducible 10-kD protein (IP-10) and monokine induced by IFN-γ (MIG) are chemokines that are secreted from immune and non-immune cells in response to IFN-γ (116), and correlate with SLE disease activity (94). CSF levels of these chemokines are elevated in NPSLE, even when compared to non-neuropsychiatric SLE patients, and fall following symptom resolution, thus suggesting utility for monitoring disease activity (83, 95). Interestingly, a study of 7 patients with lupus-related headaches showed higher CSF IP-10 levels compared to non-neuropsychiatric SLE patients, and CSF MIG levels compared to non-neuropsychiatric SLE and non-headache NPSLE patients (95). This may, again, emphasize the potential association of certain markers with specific NPSLE manifestations.

### 4.3. Novel neuroimaging studies

#### 4.3.1. Quantitative MRI studies

In contrast to cMRI, quantitative MRI techniques are sensitive to physiological and microstructural tissue changes (Table 3) and have interestingly shown such changes not only in individuals with NPSLE, but also individuals with SLE not known to have neuropsychiatric involvement.

**Table 3:** Novel neuroimaging studies in NPSLE.

DTI (117, 118)	Facilitates measurement of water molecule diffusion (MD) and the direction of diffusion (FA)
	DTI parameters (increased MD, reduced FA) provide an assessment of the microarchitectural integrity of WM tracts, which can even be compromised in areas of normal-appearing WM seen on cMRI
MTI (117, 119)	Differences in magnetic properties between protons in free water and those bound within immobile tissues produce signal changes quantitatively expressed as the MTR
	Reductions of the MTR may indicate compromised WM integrity, even in normal-appearing WM on cMRI
MRS (117, 120)	Produces spectra from nuclei including ^1^H, ^13^C, ^23^Na, and ^31^P enabling quantification of neuronal metabolites, and thus cellular function
	NAA is found within neurons and axons within GM and WM, and may indicate neuronal density, function and integrity
	Cho is a marker of cell wall integrity, and may increase in pathological WM states
	Cr levels are stable within the brain and is used as an internal reference for other neuronal metabolites
fMRI (121)	Utilizes differences in the magnetic properties between oxygenated and deoxygenated blood to assess neuronal activity
	Performed either in ‘resting state’, during various cognitive tasks
	Rs-fMRI assesses baseline cerebral activity in the absence of cognitive or sensory stimuli
	May be performed by measuring signal characteristics within a region of interest or by measuring the relationship or connectivity between spatially different brain regions
ASL (122)	Involves radiofrequency labelling of intra-arterial water protons
	Assesses cerebral perfusion by measuring signal differences between radiofrequency-labelled arterial blood water protons and cerebral tissue protons in the tissues of interest
DSC-MRI (123)	Utilizes contrast media to assess cerebral perfusion in specific regions of interest
	Increased, decreased, or even variability of perfusion parameters may indicate pathological states
NM-SPECT	Utilizes radiotracer dye to highlight cerebral blood flow and perfusion
	Typically qualitative assessment to assess for asymmetrical perfusion
	Regional changes in perfusion suggestive of pathological states
FDG-PET	Utilizes radiolabelled glucose to highlight cerebral metabolism
	Regional changes in metabolism may be indicative of microstructural damage or physiological dysfunction

ASL, arterial spin labelling; cMRI, conventional magnetic resonance imaging; Cho, choline; Cr, creatine; DSC-MRI, dynamic susceptibility contrast MRI; DTI, diffusion tensor imaging; DWI, diffusion-weighted imaging; FA, fractional anisotropy; FDG-PET, F-18 fluorodeoxyglucose positron emission tomography; fMRI; functional MRI; GM, grey matter; MD, mean diffusivity; MRS, magnetic resonance spectroscopy; MTI, magnetization transfer imaging; MTR, magnetization transfer ratio; NAA, N-acetyl aspartate; NM-SPECT, nuclear medicine single-photon emission computed tomography; Rs-fMRI; resting state fRMI; WM, white matter.

A systematic review of diffusion tensor imaging (DTI) in SLE, including 195 NPSLE and 299 SLE patients without neuropsychiatric manifestations, revealed that both groups showed abnormal DTI parameters [reduced fractional anisotropy (FA), increased mean diffusivity (MD)] in white matter (WM) regions (indicative of damage), suggesting CNS involvement even in those without neuropsychiatric complaints, although also showed the potential to distinguish both groups based on differences in these quantifiable parameters (124). Adding to this, a study of 39 NPSLE and 25 non-neuropsychiatric SLE patients revealed abnormal parameters in the corpus callosum correlated with SLE disease duration, although did not correlate with SLE disease activity nor the presence of WM hyperintensities on cMRI (125). Further studies will be needed to determine if there are any associations between DTI parameters and specific neuropsychiatric syndromes.

Studies of magnetization transfer imaging (MTI) have demonstrated reduced magnetization transfer (MT) parameters in NPSLE than non-neuropsychiatric SLE cohorts, even following adjustment for brain volume and intracranial volume, suggesting both a greater degree of microstructural parenchymal damage and cerebral atrophy in NPSLE cohorts, respectively (126, 127). Additionally, reduced MT parameters have shown associations with assessments of cognitive and psychiatric function, and correspondingly change with clinical improvement (128, 129). Furthermore, a study including 19 NPSLE patients also demonstrated changes distinguishing active from chronic stage NPSLE, thus highlighting a possible role in diagnosing and monitoring NPSLE activity and treatment adequacy (127).

MR spectroscopy has also been studied. Reduced N-acetyl choline (NAA)/creatine (Cr) ratios have been demonstrated in SLE, and are lower in NPSLE than non-neuropsychiatric SLE, including in areas of normal-appearing WM (130). Additionally, a lower NAA/Cr ratio has even been reported in active SLE compared to inactive SLE, independent of neuropsychiatric involvement, suggesting a relationship with SLE disease activity (131). Increased choline (Cho)/Cr ratios have also been demonstrated in SLE patients, which has additionally shown associations with cognitive impairment or in those with a history of neuropsychiatric involvement (120, 130, 132).

Functional MRIs (fMRI) measure blood oxygen-dependent signals that reflect neuronal activity and have facilitated identification of networks involved in different cerebral functions. Their use has also been studied in NPSLE. Resting state (Rs-) fMRI studies, performed in the absence of cognitive stimuli, have demonstrated altered brain activity and inter- and intra-network connectivity in both NPSLE and non-neuropsychiatric SLE populations, with more severe findings shown in the former, as well as associations with disease activity, duration, cognitive performance, anxiety, and depression (133–137).

Arterial spin labelling (ASL), a contrast-free MR perfusion technique, has also demonstrated changes in cerebral blood perfusion in both NPSLE and non-neuropsychiatric SLE cohorts, although again with greater abnormalities and at higher incidence in the former (138, 139).

Dynamic susceptibility contrast (DSC-) MRI, which assesses cerebral perfusion through measurement of cerebral blood volume (CBV) and cerebral blood flow (CBF) in specific regions of interest, has shown increased perfusion in normal appearing cerebral tissue of SLE cohorts (123, 140). While studies have generally demonstrated conflicting results in perfusion parameters of NPSLE cohorts (141, 142), part of which may be due to utilization of different imaging analysis protocols, such findings may also be explained by an increased variability of perfusion parameters in NPSLE, as demonstrated by a study including 24 NPSLE and 21 non-neuropsychiatric SLE patients (123). Further research with more uniform protocols, however, may better determine the role of such perfusion studies in NPSLE.

It is possible that neuroimaging findings in non-neuropsychiatric SLE populations are reflective of early or subclinical disease. More studies, however, will be needed to confirm the utility of these imaging modalities for distinguishing NPSLE from non-SLE-related psychiatric manifestations, as well as to determine the clinical implications of abnormal findings in patients with SLE in the absence of a history of neuropsychiatric manifestations.

#### 4.3.2. Nuclear medicine studies

**Figure 2:**
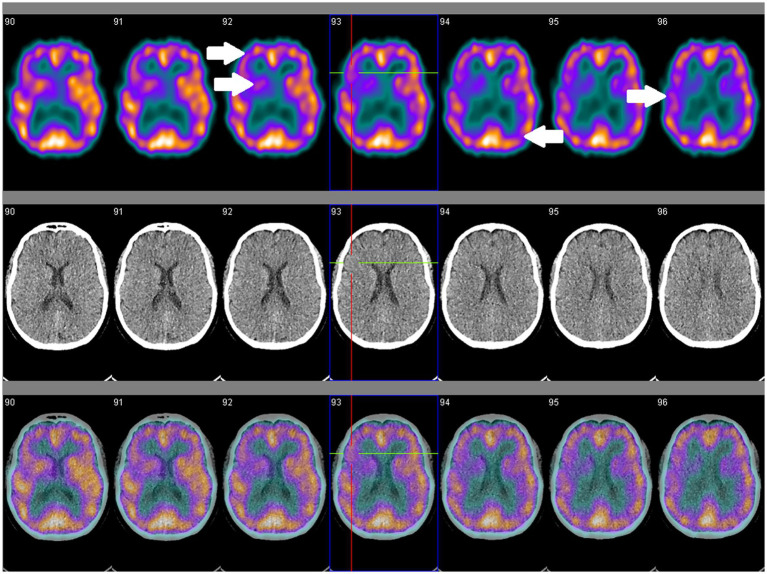
SPECT coregistered to CT (SPECT/CT) in a 56-year old female with clinically and serologically inactive SLE and no overt neuropsychiatric symptoms showing asymmetrical perfusion. Comparison between right and left sides are necessary for qualitative assessment. Focal areas of hypoperfusion (arrows) are seen in the frontal, temporal and occipital lobes, and the caudate, putamen and thalamus.

Studies of NM-SPECT have suggested greater sensitivity than cMRI for detecting cerebral involvement by SLE (143). Regional hypoperfusion has been reported in all SLE patient groups, although at higher frequency in active than inactive NPSLE or non-neuropsychiatric SLE populations, and more commonly occurs in the frontal, parietal and temporal lobes, and less commonly in the cerebellum and basal ganglia (143, 144). Two small studies reported opposing findings on the reversibility of these changes following corticosteroid treatment, although was more favorable in the study that utilized a higher treatment dose, which may suggest utility for monitoring disease activity and treatment response (145, 146). Further investigations using uniform treatment protocols may, however, be needed to better elucidate its utility in monitoring NPSLE activity. Nevertheless, a study of 66 NPSLE and 41 non-neuropsychiatric SLE patients established that concordantly normal cMRI and NM-SPECT findings were associated with non-neuropsychiatric SLE, thus suggesting that coupling these may be more useful to exclude rather than confirm NPSLE (147). Most of these studies of NM-SPECT, however, are a decade old, so future studies should consider assessing the utility of combined SPECT–CT or SPECT-MRI for attenuation correction and image co-registration in NPSLE cohorts (Figure 2).

F-18 fluorodeoxyglucose (FDG) PET on NPSLE cohorts have demonstrated various regional hyper- or hypometabolic changes, commonly in the temporal, occipital and frontal lobes, which have also shown associations with impaired memory and mood disorders (148–150). Although no associations with SLEDAI scores have been observed, serial PET imaging in a small study demonstrated normalization following improvement of neuropsychiatric symptomatology (151). Further studies will need to better determine its utility, however advances in PET have also seen the introduction of agents other than FDG, some of which may prove useful in assessing NPSLE in the future (152).

No head-to-head comparisons between these neuroimaging modalities in NPSLE have been made. It is possible that a combination of these will need to form part of an algorithm for the investigation of NPSLE.

## 5. A perfect algorithm?

Our case presentation highlights the challenges of diagnosing neuropsychiatric involvement by SLE. Notably, following our case patient’s established SLE diagnosis, during which she presented with clinical features compatible with a classification of SLE as per the EULAR/ACR 2019 Classification Criteria for SLE, she presented once with a new psychosis and once with mania, both of which are neuropsychiatric syndromes outlined in the 1999 ACR case definitions for neuropsychiatric syndromes in NPSLE, whilst on treatment for her SLE. Notably, on both occasions she appeared to have achieved and maintained a state otherwise of clinical and serological remission, having resolution of musculoskeletal symptoms, and normalization of inflammatory markers, serum complement levels and proteinuria. The question, therefore, remained as to whether these presentations were either driven by or independent of the SLE, particularly in light of the unremarkable CSF analysis and MRI findings, and the non-specific NM-SPECT findings.

**Figure 3:**
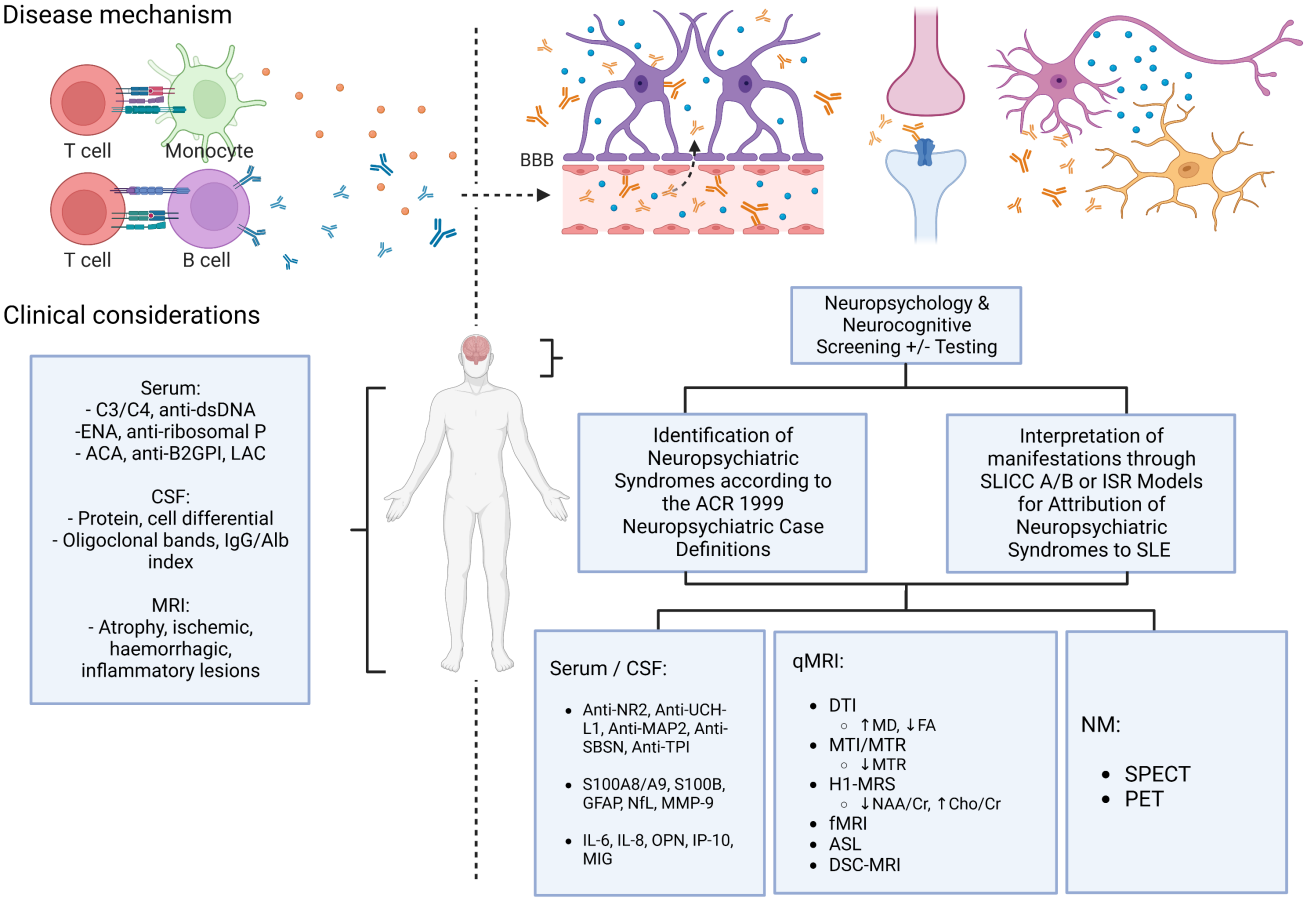
Current & proposed diagnostic algorithm in suspected NPSLE. ACA, anti-cardiolipin antibodies; ACR, American College of Rheumatology; anti-β2GPI, anti-beta-2-glycoprotein I; ASL, arterial spin labelling; BBB, blood–brain barrier; Cho, choline; Cr, creatine; DSC, dynamic susceptibility contrast; DTI, diffusion tensor imaging; ENA, extractable nuclear antigens; FA, fractional anisotropy; fMRI, functional MRI; H1-MRS, magnetic resonance spectroscopy; ISR, Italian Society of Rheumatology; LAC, lupus anticoagulant; MD, mean diffusivity; MTI/MTR, magnetization transfer imaging/ratio; NAA, N-acetyl aspartate; NM, nuclear medicine; qMRI, quantitative MRI; SLICC, Systemic Lupus International Collaborating Clinics.

Does an algorithm exist to better classify NPSLE? As described above, there are many tools that do not typically form part of the routine assessment of SLE patients, however may help attribute neuropsychiatric phenomena to SLE with better specificity (Figure 3). Neuropsychological screening tools show utility for detecting mood or cognitive disorders which may often be unapparent without structured assessment. Consideration of detected neuropsychiatric phenomena and interpretation of their relationship to SLE through the Italian Society of Rheumatology or SLICC attribution models may help better determine their significance, particularly in the case of non-specificity of other conventional markers of SLE activity. Additionally, studies of more novel SLE investigations have shown the promise of a number of different serological and CSF markers and neuroimaging tools, which may hopefully show more concrete evidence for identifying NPSLE in the future.

Confusingly, what is evident with the collective work on these novel markers and neuroimaging studies is that abnormalities are not limited to those classified to NPSLE and may also be seen in those without reported neuropsychiatric manifestations. Part of this may be due to the variable SLE classification criteria utilized by the different studies, as well as variations in the definitions of what constitutes a classification of NPSLE – and therefore ongoing studies of these individual markers and modalities using more stringent and uniform definitions for NPSLE will be needed to better establish their use in the classification of NPSLE in the future. Alternatively, the presence of such abnormalities even in those classified as non-neuropsychiatric SLE patients may also argue for establishing or revising pre-existing algorithms for classifying NPSLE, and raises the question as to whether all new diagnoses of SLE, independent of neuropsychiatric phenomena, require screening with novel serological or CSF markers and neuroimaging methods for prognostication of neuropsychiatric involvement.

## 6. Conclusion

Despite current advancements in the knowledge of NPSLE, gaps in investigation algorithms still hinder its diagnosis at various stages of patient work up – including poor recognition of neuropsychiatric syndromes, unremarkable serological and CSF markers and non-specific conventional neuroimaging study results. Furthermore, research efforts have also been limited due to the lack of standardized classification criteria or definition of what constitutes a diagnosis of NPSLE. This conundrum has prompted consideration of novel serological, CSF and neuroimaging studies, which may narrow these gaps and distinguish neuropsychiatric involvement from confounding non-immunological neuropsychiatric disease processes, such as steroid-induced psychosis or schizophrenia, with better specificity.

While individual strategies have shown utility in distinguishing NPSLE from other SLE and non-SLE cohorts – which is one of the major challenges of NPSLE – a combination of investigations may better assist in diagnosis and monitoring. Further studies will be needed to better determine the best combination of modalities, which will also have to be weighed against accessibility, safety and the experience needed with these strategies. Likewise, treatment algorithms for NPSLE are similarly not well-defined and are at best derived from small RCTs and cohort studies, and thus more work will also be needed to determine better therapeutic strategies in NPSLE, particularly given the growing experience with newer treatments such as belimumab and anifrolumab.

## Author contributions

All authors have substantially contributed to the conception of this study, acquisition and analysis of data, and preparation of this manuscript. JE was involved in the process of the manuscript write up. MWL, SS, SG, and LG were involved in manuscript review and revisions. All authors contributed to the article and approved the submitted version.

## Conflict of interest

The authors declare that the research was conducted in the absence of any commercial or financial relationships that could be construed as a potential conflict of interest.

## Publisher’s note

All claims expressed in this article are solely those of the authors and do not necessarily represent those of their affiliated organizations, or those of the publisher, the editors and the reviewers. Any product that may be evaluated in this article, or claim that may be made by its manufacturer, is not guaranteed or endorsed by the publisher.
